# Associations of Bovine Fetal Adipose Tissue-Derived Mesenchymal Stem/Stromal Cells and Germ Cell Marker Expression in Testicular Organoids

**DOI:** 10.3390/ani16182933

**Published:** 2026-09-18

**Authors:** Jahaira Cortez, Víctor H. Parraguez, Carlos Díaz, Oscar A. Peralta

**Affiliations:** 1 Faculty of Veterinary and Animal Sciences, University of Chile, Santa Rosa 11735, Santiago 8820808, Chile; jahazu86@gmail.com (J.C.); vparragu@uchile.cl (V.H.P.); 2 Doctorate Program of Forestry, Agriculture, and Veterinary Sciences (DCSAV), University of Chile, Santa Rosa 11315, Santiago 8820808, Chile; 3 Programa de Doctorado en Ciencias, Escuela Internacional de Doctorado de la UNED (EIUNED), Bravo Murillo 38, 28015 Madrid, Spain; cdiab@uc.cl; 4 Escuela de Medicina Veterinaria, Facultad de Agronomía y Sistemas Naturales, Facultad de Ciencias Biológicas y Facultad de Medicina, Pontificia Universidad Católica de Chile, Vicuña Mackenna 4860, Santiago 7820436, Chile

**Keywords:** mesenchymal stem/stromal cells, 3D culture, testicular organoid, germ cell markers

## Abstract

Organoids serve as three-dimensional structures that offer spatial support and mimic cellular niches for experimental studies. The spatial and temporal association of fetal bovine adipose tissue-derived mesenchymal stem/stromal cells (AT-MSCs) with the expression of germ cell (GC) markers was evaluated in testicular organoids (TOs+AT-MSCs) under the effect of retinoic acid and testosterone (0.2, 2 and 20 µM). TOs+AT-MSCs were developed by the co-culture of Leydig, Sertoli, and peritubular myoid cells and PKH26+AT-MSCs and were analyzed for the expression of DAZL, STRA8 and UCHL1. TOs+AT-MSCs treated with 1 µM of retinoic acid and 2 or 20 µM of testosterone maintained their diameter after day 7 of culture. Pearson correlation coefficients (PCCs) of PKH26 and DAZL were higher (*p* < 0.05) at days 7 and 21 in TOs+AT-MSCs treated with 1 µM retinoic acid and 0.2 or 2 µM of testosterone, respectively. PCCs of PKH26 and STRA8 or UCHL1 were higher (*p* < 0.05) at days 14 and 21 in TOs+AT-MSCs treated with 1 µM retinoic acid and 0.2 or 2 µM of testosterone. Thus, exposure to 1 µM of retinoic acid and 2 µM of testosterone resulted in a similar diameter with respect to the control and spatial and temporal association of AT-MSCs and DAZL, STRA8 and UCHL1 markers in TOs+AT-MSCs.

## Simple Summary

Testicular organoids (TOs) are small, three-dimensional laboratory models that mimic the structure and functions of the testis. In this study, we investigated whether mesenchymal stem/stromal cells obtained from fetal bovine adipose tissue (AT-MSCs) were associated with the presence of germ cell (GC)-specific markers in TOs. TOs were composed of testicular cells (Leydig, Sertoli, and peritubular myoid cells) and fluorescently labeled AT-MSCs and treated with retinoic acid and different concentrations of testosterone for 21 days. We found that treatment with retinoic acid combined with moderate concentrations of testosterone helped maintain TO size and increased the association of AT-MSCs with markers of GCs. Overall, the combination of retinoic acid and testosterone influenced the location and timing of GC marker expression in TOs.

## 1. Introduction

Mesenchymal stem/stromal cells (MSCs) are adult progenitor cells that can both self-replicate and differentiate into different types of connective tissue, specifically, bone, fat, and cartilage [1,2]. MSCs are considered a promising therapeutic tool since they are relatively easy to isolate, possess the ability to differentiate into various cell types, and exhibit trophic effects that contribute to tissue regeneration [3,4,5]. A significant benefit of MSCs is their potential for being used in autologous or allogenic cell therapies without the ethical considerations associated with embryonic stem cells (ESCs) [4,5,6]. The therapeutic potential of MSCs has been supported by numerous preclinical and clinical trials across a spectrum of diseases, including reproductive disorders in both females and males [3,4,5,6]. In male infertility, MSC therapy is being explored as a treatment for azoospermia and oligozoospermia [3]. Additionally, exosomes produced by MSCs have shown the ability to support sperm production in animal models of infertility [7], and studies suggest that MSCs can even develop into germ cell-like cells [8,9,10], pointing towards their potential in treating infertility.

The testicular niche represents a specific anatomical microenvironment essential for the differentiation and development of spermatogonial stem cells (SSCs) [11]. This niche is characterized by intricate interactions with surrounding cellular components, including Sertoli, Leydig, peritubular, endothelial, and nerve cells [12,13], and is structurally supported by the extracellular matrix (ECM). A detailed understanding of this complex microenvironment is critical for elucidating the key regulatory factors and signaling pathways governing germ cell development, maturation, and function. This knowledge can facilitate the development of targeted therapeutic interventions for infertility, such as optimizing in vitro culture systems, enhancing germ cell (GC) viability and differentiation, and exploring innovative regenerative approaches such as GC transplantation [7].

The advent of 3D cultures, particularly organoids, in recent years has provided essential in vitro platforms for cellular modeling [12,13]. A key objective of organoid culture is the recapitulation of the cellular niche or microenvironment, thereby exposing cultured cells to conditions that closely approximate those of native tissue [14]. In contrast to conventional 2D culture systems, which lack the inherent structural and interactive complexity of in vivo contexts, 3D cultures facilitate functional differentiation, enhancing their physiological relevance [15]. Irrespective of the specific organoid type, the typical core constituents comprise: (1) a basement membrane extract or hydrogel, such as Matrigel; (2) tissue-specific stem or progenitor cells; and (3) a culture medium supplemented with growth factors designed to mimic in vivo signaling pathways [16,17,18]. These components collectively support cellular development and organization in a manner that more accurately reflects the structural and functional attributes of natural tissues.

While organoid technology has seen a surge in interest over the last 10 years, testicular organoids (TOs) are a more recent area of significant investigation [15]. Initial studies showed that TOs could be generated from primary adult and pubertal human testicular cells, notably, capable of self-organization without a structural scaffold [19]. Further advancements in 2017 led to the development of TOs from rat testicular cells and human immortalized Leydig and Sertoli cells [20,21], which were functional, secreting inhibin B, testosterone, and cytokines, and showing Sertoli cells forming tight junctions and potentially supporting GC development. Since then, the successful creation of TOs has been reported in various species, including pigs, primates, and mice [13,22,23]. Notably, our research group recently reported the development of the first bovine TOs using a protocol that includes isolation of Leydig, Sertoli, and peritubular myoid cells and culture in ultra-low-attachment (ULA) plates [24]. These bovine TOs displayed a specific spatial organization of the different testicular cell types and demonstrated the production of testosterone, with levels increasing over a 27-day culture period [24]. Furthermore, we sought to evaluate the co-culture of adipose tissue-derived MSCs (AT-MSCs) with TOs and to investigate the potential impact of AT-MSCs on testicular cell populations conforming to a testicular niche [25]. PKH26-labeled AT-MSCs were observed during the whole culture period, displaying a widespread distribution in TOs+AT-MSCs. In contrast to AT-MSCs, which were located in the center area of TOs+AT-MSCs, Leydig cells formed a compact peripheral layer [25]. In comparison, Sertoli cells were observed within the central area, forming clusters of cells and revealing some compartmentalization in relation to AT-MSCs [25]. Similarly, peritubular myoid cells were detected in the central region of TOs+AT-MSCs in decreasing number in comparison to AT-MSCs [25].

Due to their in vitro nature, the growth and maintenance of organoid tissue can be observed directly by time-lapse microscopy. However, cell-tracking approaches have only scarcely been applied to organoids thus far. Moreover, in the case of Tos, there is still a challenge of distinguishing true germ cell-like differentiation from the non-specific expression of germ cell-related markers in MSCs.

The therapeutic versatility of MSCs has not yet been fully translated into in vitro models, potentially due to gaps in our understanding of their mechanisms. Addressing the critical need for innovative biotechnological solutions for infertility, TOs emerge as a promising in vitro system [20,21,22]. MSCs offer therapeutic potential through their differentiation capacity, immunomodulatory and paracrine actions, and antioxidative properties that promote tissue repair. Consequently, the aim of the present study was to evaluate the spatial and temporal association of fetal bovine AT-MSCs with the expression of GC markers in TOs under the exogenous effect of retinoic acid and testosterone.

## 2. Materials and Methods

### 2.1. Ethics Statement

All experimental procedures were approved and were performed in accordance with the guidelines and regulations of the Bioethical Committees of the Faculty of Veterinary and Animal Sciences at the University of Chile (Certificate N° 19266-VET-UCH) and the Pontifical Catholic University of Chile (Certificate N° 230315003).

### 2.2. Study Design

**Figure 1:**
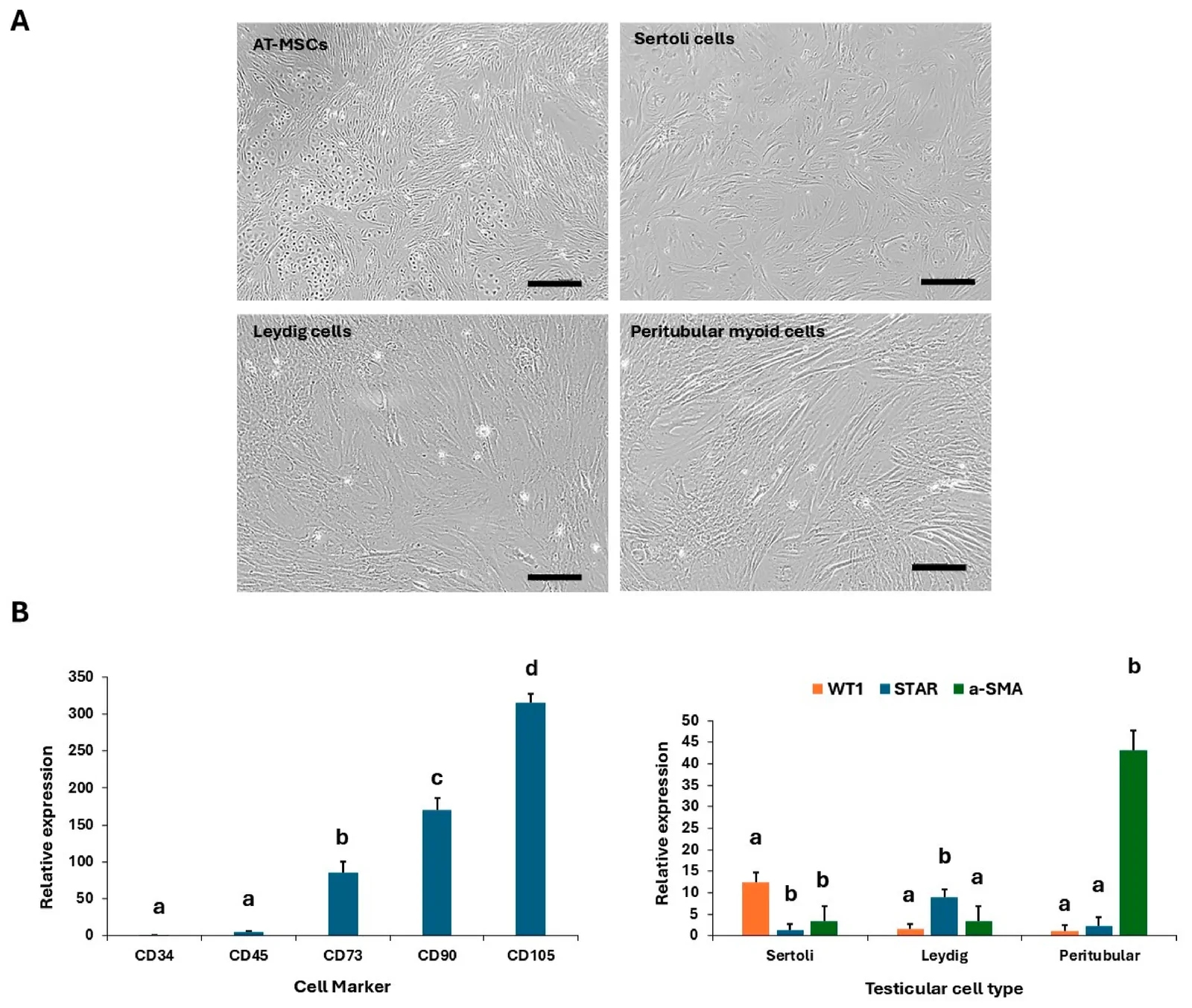
Morphology and cell marker gene expression profile in bovine AT-MSCs and testicular cells used for TO production. (**A**) Subpopulations of AT-MSCs displayed characteristic polygonal and fibroblast morphology. Leydig and peritubular myoids cells showed an epithelioid and slightly elongated shape. Sertoli cells displayed a more rounded or cubical morphology. Scale bar: 500 μm. (**B**) AT-MSCs expressed high (*p* < 0.05) mRNA levels of MSC markers CD73, CD90, and CD105 and low (*p* < 0.05) mRNA levels of hematopoietic markers CD34 and CD45. Gene expression levels of WT1, STAR and αSMA were detected in all testicular cell cultures, with respective higher (*p* < 0.05) mRNA levels in Sertoli, Leydig and peritubular myoid cells. Superscript (a, b, c, d) indicates significant (*p* < 0.05) difference in relative expression between MSC markers ((**B**), left) and relative expression between testicular cell markers for each testicular cell type ((**B**), right).

**Figure 2:**
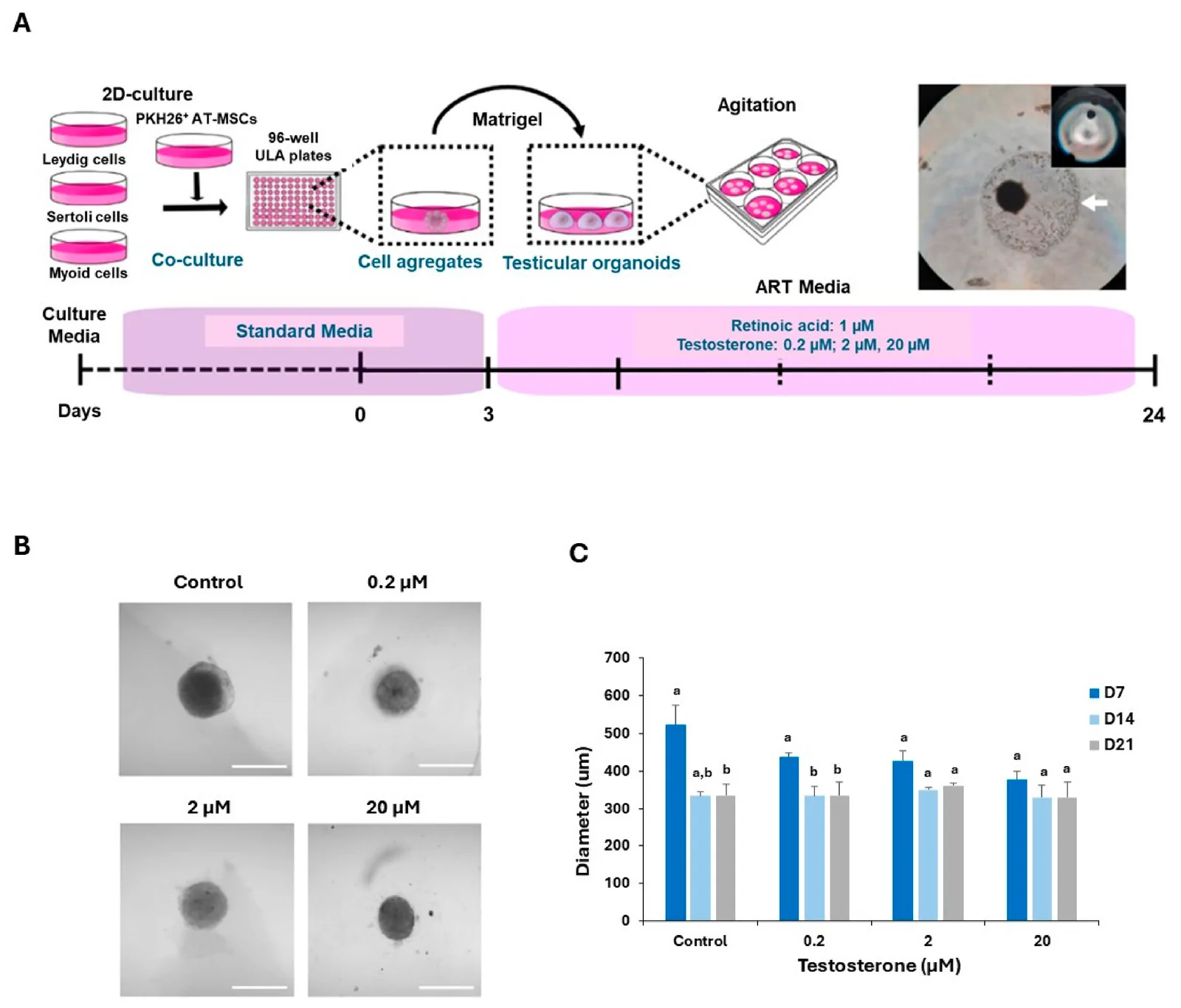
Schematic diagram depicting the main steps for bovine TOs+AT-MSCs production and morphological and diameter comparison in TOs+AT-MSCs treated with retinoic acid and testosterone. (**A**) AT-MSCs labelled with PKH26 (PKH26+) were cocultured with testicular cells in 96-well ultra-low adherence (ULA) plates to produce TOs+AT-MSCs. Bright-field image displays a spherical TO after 7 days of culture in Matrigel (white arrow). (**B**) TOs+AT-MSCs cultured in the presence of 1 µM of retinoic acid and 0.2, 2 or 20 µM of testosterone displayed an outer layer of cells surrounding the central structure of the organoid. Scale bar: 500 μm. (**C**) Treatment with 1 µM of retinoic acid and 2 or 20 µM of testosterone resulted in a similar diameter in TOs+AT-MSCs at days 7, 14 and 21 of culture. Superscript (a, b) indicates significant (*p* < 0.05) difference in diameters between days of culture (7, 14 and 21, after Matrigel seeding) for each testosterone concentration. Three TOs were analyzed for diameter for each day and treatment.

Bovine fetuses (n = 9) and adult testis (n = 9) were collected from a local abattoir, following university biosafety guidelines. AT-MSCs were collected and pooled from three bovine fetuses. Testicular cells were isolated and pooled from three adult bull testis. Each pool represented a biological replicate, and experiments and analyses were performed thrice in each pool. Pooling tissue samples was performed to reduce individual biological variation. All cell types were characterized according to the gene expression of specific markers for MSCs (−CD34, −CD45, +CD73, +CD90 and +CD105), SCs (+WT1), Leydig cells (+STAR), and peritubular myoid cells (+αSMA) using quantitative PCR (Q-PCR) (Figure 1). AT-MSCs and testicular cells were co-suspended at a total concentration of 1 × 10^6^ cells (1:1 concentration; 5 × 10^5^ pool of testicular cells and 5 × 10^5^ of AT-MSCs) per well in 100 μL of standard culture medium in each of a 96-well Ultra-Low Attachment (ULA) U-shaped bottom plate (Corning, NY, USA) to form TOs [23,24] (Figure 2). TOs were cultured (3 replicates, and each replicate corresponded to TOs derived from one cell pool) in DMEM F12 supplemented with 10% FBS, 100 IU/mL penicillin, 100 μg/mL streptomycin and 100 μg/mL amphotericin B. After 3 days of culture, the cellular aggregates were incubated with 40 μL of Matrigel (Corning, NY, USA) for 10–20 min at 38 °C to facilitate solidification. Subsequently, TOs (TOs with AT-MSCs) were transferred to 6 cm diameter ULA plates and were divided into four experimental conditions consisting of (1) Control in standard culture medium, or three treatments, each with 1 µM of retinoic acid and either (2) 0.2 µM of testosterone; (3) 2 µM of testosterone; or (4) 20 µM of testosterone. TOs were collected at days 7, 14 and 21 of culture and analyzed by phase contrast microscopy for morphological characteristics, and examined for diameter and confocal immunofluorescence analyses of DAZL, STRA8 and UCHL1 immunodetection (3 TOs for each day and treatment) and colocalization with PKH26 (10–12 TOs for each day and treatment).

### 2.3. Isolation of Bovine AT-MSCs

Bovine fetal MSCs were obtained from AT isolated from the abdominal omentum (8–9 months of gestation), following a protocol previously reported [24,25]. Briefly, AT was incubated in an enzymatic solution containing 0.5% type 1 collagenase (Sigma-Aldrich, Saint Louis, MO, USA) diluted in balanced Hanks’ saline solution (HBSS) (1 mL/g of AT) for manual disaggregation. The cell pellet was resuspended in expansion medium (DMEM supplemented with 10% fetal bovine serum (FBS), 100 μg/mL streptomycin, 100 U/mL penicillin, and 0.25 mg/mL amphotericin B) and transferred to T75 culture bottles (Falcon, Corning, NJ, USA) at 38 °C in a humid atmosphere with 5% CO_2_. The non-adherent cells were removed by changing the culture medium after 2 days.

### 2.4. AT-MSC Membrane Labeling with PKH26

AT-MSCs (5 × 10^5^ for each TO) were collected, as previously described, and labeled with PKH26 dye, following the manufacturer’s instructions (PKH26GL, Sigma-Aldrich, Saint Louis, MO, USA), to track their localization in the TOs. Briefly, the cells were resuspended and then centrifuged (400× *g*) for 5 min to obtain a loose pellet. Subsequently, the supernatant was removed, and 1 mL of diluent C was added to the cell pellet, which was gently pipetted to ensure complete dispersion. A dye solution was prepared by adding 4 µL of PKH26 to 1 mL of Diluent C. Quickly, 1 mL of cell suspension was added to 1 mL of dye solution and mixed immediately. The stained cells were incubated for 1 to 5 min, followed by the addition of 2 mL of serum and an additional incubation for 1 min. Cells were then centrifuged at 400× *g* for 10 min at 20–25 °C, and the supernatant was carefully removed. Subsequently, they were resuspended in 10 mL of culture medium, transferred to a new tube, and centrifuged two more times at 400× *g* for 5 min each with culture medium to ensure the removal of unbound dye.

### 2.5. Bovine Testis Collection and Preparation

A total of 9 bovine adult testis (2 years of age) were used to obtain testicular cells. Testis were initially washed with calcium-magnesium-free HBSS (Hyclone Laboratories, Logan, UT, USA) supplemented with 100 μg/mL streptomycin and 100 U/mL penicillin (HBSS P/E). Subsequently, the tunica albuginea was removed, and the parenchyma was carefully dissected for collection of samples from the upper, medium and lower sections of the testis. Approximately 15 g of testicular tissue was cut into fragments. The tissue fragments were then suspended in HBSS P/E and allowed to settle by gravity, after which the supernatant containing GCs was discarded to separate the testicular cells from the extracellular matrix.

#### Isolation of Bovine Sertoli and Peritubular Myoid and Leydig Cells

Testicular cell isolation from bovine testis was performed following a previously established protocol [24,25,26]. Briefly, testicular tissue was incubated in an enzymatic solution composed of HBSS P/S, 2.5% trypsin, 1% type IV collagenase, and 1 mg of DNase. After a series of gravity sedimentation steps, peritubular myoid cells, Leydig cells and Sertoli cells were sequentially isolated and cultured in MEM supplemented with 10% FBS, 100 U/mL penicillin, 100 μg/mL streptomycin, and 100 μg/mL amphotericin B. Subsequently, Leydig cell fraction was enriched using a discontinuous 5-layer Percoll gradient, comprising concentrations of 21, 26, 34, 60 and 100% in PBS. Thereafter, cells were plated and cultured in DMEM:F12 with P/S and supplemented with 10% fetal bovine serum (FBS). The culture medium was replaced every 2–3 days.

### 2.6. Quantitative PCR Analysis of Cell-Specific Markers in Bovine Testicular Cells

The quantification of mRNA levels of endogenous genes GAPDH and β-ACTIN, MSC genes (CD73, CD90 and CD105), and hematopoietic genes (CD34 and CD45), as well as testicular cell markers αSMA (peritubular myoid cells), WT1 (Sertoli cells), and STAR (Leydig cells), was performed using quantitative real-time PCR (Q-PCR) (Table 1). Total RNA was extracted from the TOs using the GeneJet RNA purification kit (Thermo Fisher Scientific, MA, USA), following the manufacturer’s instructions. Total mRNA was quantified using a Qubit 3.0 Fluorometer (Thermo Fisher, Carlsbad, CA, USA). Genomic DNA was removed using a DNAse I kit (Thermo Scientific, Carlsbad CA, USA). cDNA synthesis and amplification were performed using the Affinity Script qPCR cDNA Synthesis Kit (Agilent Technologies, Santa Clara, CA, USA) and a TC1000-G thermocycler (SciLogex, Rocky Hill, CT, USA). The Q-PCR reaction utilized the Brilliant SYBR Green qPCR Master Mix (Agilent Technologies, Santa Clara, CA, USA) and an Eco Real-Time PCR System (Illumina, San Diego, CA, USA). cDNA amplification was carried out for 40 cycles, and the relative expression analysis was performed using the ΔΔCt method. Thermal cycling conditions were 95 °C for 10 min, followed by 40 repetitive cycles at 95 °C for 30 s, and 60 °C for 1 min. To ensure that amplicons were generated from mRNA and not from genomic DNA amplification, control samples without reverse transcriptase (NRT) were included. Amplifications were followed by dissociation (melting) curves to ensure the specificity of the primers. Primers were designed using the National Center for Biotechnology Information (NCBI) database to find the required nucleotide sequences. Subsequently, the NCBI primer design tool was used, and the performance of the primers for (Q-PCR) was verified using the Primer Premier software (version 6.0). The equivalence of amplification efficiencies among all primer–probe sets was confirmed using serial 3-fold dilutions of PBL cDNA. In each experiment, the amount of gene expression was recorded as CT values that corresponded to the number of cycles where the fluorescence signal could be detected above a threshold value. The CT averages for each biological replicate were calculated and transformed into relative values denominated quantity (Q) through the ∆∆CT formula. Then, the relative quantification in the expression of target genes for each sample was estimated as the quotient between the Q value of the target gene and a normalization factor (NF), which was calculated based on the geometric mean of housekeeping gene Q values. Despite multiple testicular cell types being analyzed, β-ACTIN and GAPDH (glyceraldehyde 3-phosphate dehydrogenase) were selected as housekeeping genes based on previous analyses from our laboratory that showed high stability [6,10,24].

### 2.7. Three-Dimensional Co-Culture of AT-MSCs and Bovine Testicular Cells

AT-MSCs and testicular cells were co-suspended at a total concentration of 1 × 10^6^ cells (1:1 concentration; 5 × 10^5^ pool of testicular cells (equal proportion) and 5 × 10^5^ of AT-MSCs) per well in 100 μL of standard culture medium in each of a 96-well Ultra-Low Attachment (ULA) U-shaped bottom plate (Corning, Corning, NY, USA) [24,25,27] (Figure 2A). The standard culture medium consisted of DMEM-F12 supplemented with 10% fetal bovine serum (FBS) (Gibco, Grand Island, NY, USA), 100 U/mL penicillin, 100 μg/mL streptomycin, and 100 μg/mL amphotericin B. After 3 days of culture, the cellular aggregates were incubated with 40 μL of Matrigel (Corning, Corning, NY, USA) for 10–20 min at 38 °C to facilitate solidification. Subsequently, TOs (TOs with AT-MSCs) were transferred to 6 cm diameter ULA plates and were divided into four experimental conditions consisting of (1) Control in standard culture medium, or three treatments, each with 1 µM of retinoic acid and (2) 0.2 µM of testosterone; (3) 2 µM of testosterone; or (4) 20 µM of testosterone. TOs were maintained with a shaker at 36 °C under a humid atmosphere with 5% CO_2_ for an additional 21 days (total of 24 days). The culture medium was changed completely every two days. The cellular aggregates were evaluated using phase contrast microscopy, and the TO formation efficiency was calculated by dividing the number of TOs by the number of seeded plates. In addition, samples were collected on days 7, 14 and 21 (after Matrigel seeding) to assess the diameter and to evaluate the expression of GC markers through confocal immunofluorescence analysis (Table 1).

### 2.8. Testicular Organoid Whole-Mounting Staining

The cellular distribution of Leydig cells, Sertoli cells, peritubular myoid cells, and AT-MSCs in TOs was analyzed using confocal immunofluorescence, following a previously established protocol [24,25,27]. Briefly, TOs were fixed overnight in 4% paraformaldehyde at 4 °C. Subsequently, antigen retrieval was performed by incubating the samples in a 10 mM sodium citrate aqueous solution containing 0.05% Tween-20 (Merck, Frankfurt, Germany) at pH 6 for 15 min at 95 °C in an electric steamer bowl. After cooling down for 15–30 min, the samples were washed three times with PBS (phosphate-buffered saline) containing 0.1% Tween-20. Permeabilization of the samples was carried out using Triton X-100 (Merck, Frankfurt, Germany) in 1X PBS for 2 h at room temperature. To block non-specific binding, the samples were incubated with 2% bovine serum albumin (BSA) in PBS for 1 h at room temperature. Immunodetection of the target proteins was performed by incubating the samples with primary antibodies diluted in 2% BSA (Table 2) for 4 days at 4 °C. Following primary antibody incubation, the samples were washed three times with 0.1% Tween in PBS. Subsequently, the samples were incubated with secondary antibodies diluted in 2% BSA for 2 days at 4 °C. Finally, the samples were counterstained with Pure Blu DAPI Nuclear Staining Dye (BioRad, Hercules, CA, USA) to visualize the cell nuclei. Immunofluorescence analysis of DAZL, STRA8 and UCHL1 in TOs+AT-MSCs included the following controls: (i) replacement of the primary antibody with non-immune serum and (ii) omission of both primary and secondary antibodies.

### 2.9. Digital Image Acquisition and Processing

Digital images were acquired using a phase contrast microscope connected to a digital camera (Motic, Xiamen, China). Confocal images of TOs+AT-MSCs were taken with a Zeiss LSM 700 confocal microscope (Carl Zeiss, Oberkochen, Germany) equipped with FV10-ASW 4.2 software (Olympus Corporation, Tokyo, Japan), and digital 3D images were analyzed with ImageJ software (v1.52a, National Institute of Health, Bethesda, MD, USA). The diameter determination of TOs+AT-MSCs included both branched and unbranched areas and was determined using ImageJ. For each treatment group, random confocal images were obtained from 3 TOs, using a multi-immersion 25× or oil immersion 40× objective for single-photon imaging. The acquisition mode settings were as follows: scan mode = frame, frame size = 1024 × 1024, line step = 2, bidirectional scanning, speed = 7, averaging number = 1, bit depth = 12 and bidirectional scanning. The z-stack mode was activated, and 26 layers (z-projection) of each image were scanned. Since TOs were considered large organoids, the tile scan was activated for image collection.

### 2.10. Colocalization of PKH26 and DAZL, STRA8 and UCHL1

TOs were collected at days 7, 14 and 21 of culture and analyzed by confocal immunofluorescence of PKH26 and DAZL, STRA8 or UCHL1. Image analysis was performed blinded for treatment and time point, with files coded prior to quantification. Single-stain and unstained controls were imaged under identical settings to assess spectral bleed-through, cross-talk, and autofluorescence, using sequential channel acquisition where needed. Colocalization between PKH26 (red channel) and each germ cell marker (DAZL, STRA8, or UCHL1; green channel) was quantified using Pearson’s correlation coefficient (PCC) using the Coloc2 method (ImageJ version 1.52a). PCCs were calculated from full three-dimensional z-stacks (26 layers). ROIs were drawn around 10–12 distinct separate TOs for each experimental condition (Control, 0.2, 2, and 20 µM) and culture time point (day 7, 14, and 21). PCCs were calculated on a pixel-by-pixel basis between red and green channel intensities within the area of each image, according to the standard formula: $r=\sum [(\mathrm{Ri}-\bar{R})(\mathrm{Gi}-\bar{G})]/\surd \left[\sum {\left(\mathrm{Ri}-\bar{R}\right)}^{2}\sum {\left(\mathrm{Gi}-\bar{G}\right)}^{2}\right]$, where Ri and Gi are the intensity values of the red and green channels at pixel i, and $\bar{R}$ and $\bar{G}$ are their respective mean intensities. PCC values range from −1 (perfect exclusion) to +1 (perfect colocalization), with values near 0 indicating no linear relationship between the two signals.

### 2.11. Statistical Analysis

Statistical analyses were performed on cell-specific marker relative expression (Q/NF), TOs+AT-MSCs diameters and PCCs of PKH26 and DAZL, STRA8 or UCHL1 in the control and TOs+AT-MSCs using the Info Stat software (Version 2017, Cordova, Argentina). Each experimental setup was repeated at least three times. The statistical models included dependent variables (Q/NF, TO diameter and PCC) and independent variables (Marker expression, cell type, treatments and time). The normality of data was evaluated using the Shapiro–Wilk method. Means values of relative expression values for each replicate were analyzed by two-way ANOVA. Diameters of the control and TOs+AT-MSCs treated with 1 µM of retinoic acid and 0.2, 2 or 20 µM of testosterone were compared between days of culture (7, 14 and 21) using Tukey’s multiple comparison test (*p* < 0.05). PCCs for PKH26 and DAZL, STRA8 or UCHL1 in the control and TOs+AT-MSCs treated with 1 µM of retinoic acid and 0.2, 2 or 20 µM of testosterone were compared between days of culture (7, 14 and 21) using the Fisher multiple comparison test (*p* < 0.05).

## 3. Results

### 3.1. Cell-Specific Marker Analysis of Bovine AT-MSCs, Leydig, Sertoli and Peritubular Myoid Cells

In order to determine the composition of the testicular cell populations used for TO formation, primary cell cultures were analyzed by using phase contrast microscopy and Q-PCR. AT-MSCs displayed subpopulations of polygonal and fibroblast morphology (Figure 1A). All testicular cells were plastic adherent, and while Leydig and peritubular myoids cells showed an epithelioid and slightly elongated form, Sertoli cells displayed a more rounded or cubical shape (Figure 1A). AT-MSCs expressed high (*p* < 0.05) mRNA levels of MSC markers CD73 (85.3-fold relative to CD34), CD90 (170.3-fold relative to CD34), and CD105 (315.4-fold relative to CD34) and low (*p* < 0.05) mRNA levels of hematopoietic markers CD34 (1-fold) and CD45 (4.5-fold relative to CD34) (Figure 1B). Gene expression levels of STAR, WT1 and αSMA were detected in all testicular cell cultures (Figure 1B). However, levels of WT1 (12.3-fold), STAR (8.9-fold) and αSMA (43.1-fold) were higher (*p* < 0.05) in respective Sertoli, Leydig and peritubular myoid cells.

### 3.2. Determination of the Effect of Retinoic Acid and Testosterone Supplementation on Diameters of TOs+AT-MSCs During a 21-Day Culture Period

The formation efficiency of TOs was 100% at 24 h. TOs+AT-MSCs cultured in the presence of 1 µM of retinoic acid and 0.2, 2 and 20 µM of testosterone displayed an outer layer of cells surrounding the central structure of the organoid (Figure 2A). Under these conditions, TOs+AT-MSCs displayed diameters that fluctuated between 266 and 601 μm (Figure 2B). The diameters of TOs+AT-MSCs (control) were higher (*p* < 0.05) on day 7 (523 ± 51.3 µm) compared to day 14 (334 ± 11.3 µm) and day 21 (335 ± 29.7 μm). Similarly, the diameters of TOs+AT-MSCs treated with 1 µM of retinoic acid and 0.2 µM of testosterone were higher (*p* < 0.05) on day 7 (438 ± 9 μm) compared to day 14 (334 ± 24.3 μm) and day 21 (335 ± 36.5 μm). In comparison, the diameters of TOs+AT-MSCs treated with 1 µM of retinoic acid and 2 μM of testosterone were not different between days of culture (day 7, 427 ± 27 μm; day 14, 349 ± 8.6 μm; day 21, 361 ± 5.7 μm). Similarly, the mean diameters of TOs+AT-MSCs treated with 1 µM of retinoic acid and 20 μM of testosterone were not different between days of culture (day 7, 379 ± 21.7 μm; day 14, 330 ± 30.6 μm; day 21, 330 ± 40 μm).

### 3.3. Colocalization of PKH26-Positive AT-MSCs and Expression of DAZL, STRA8 and UCHL1 in TOs+AT-MSCs Treated with Retinoic Acid and Testosterone During a 21-Day Culture Period

**Figure 3:**
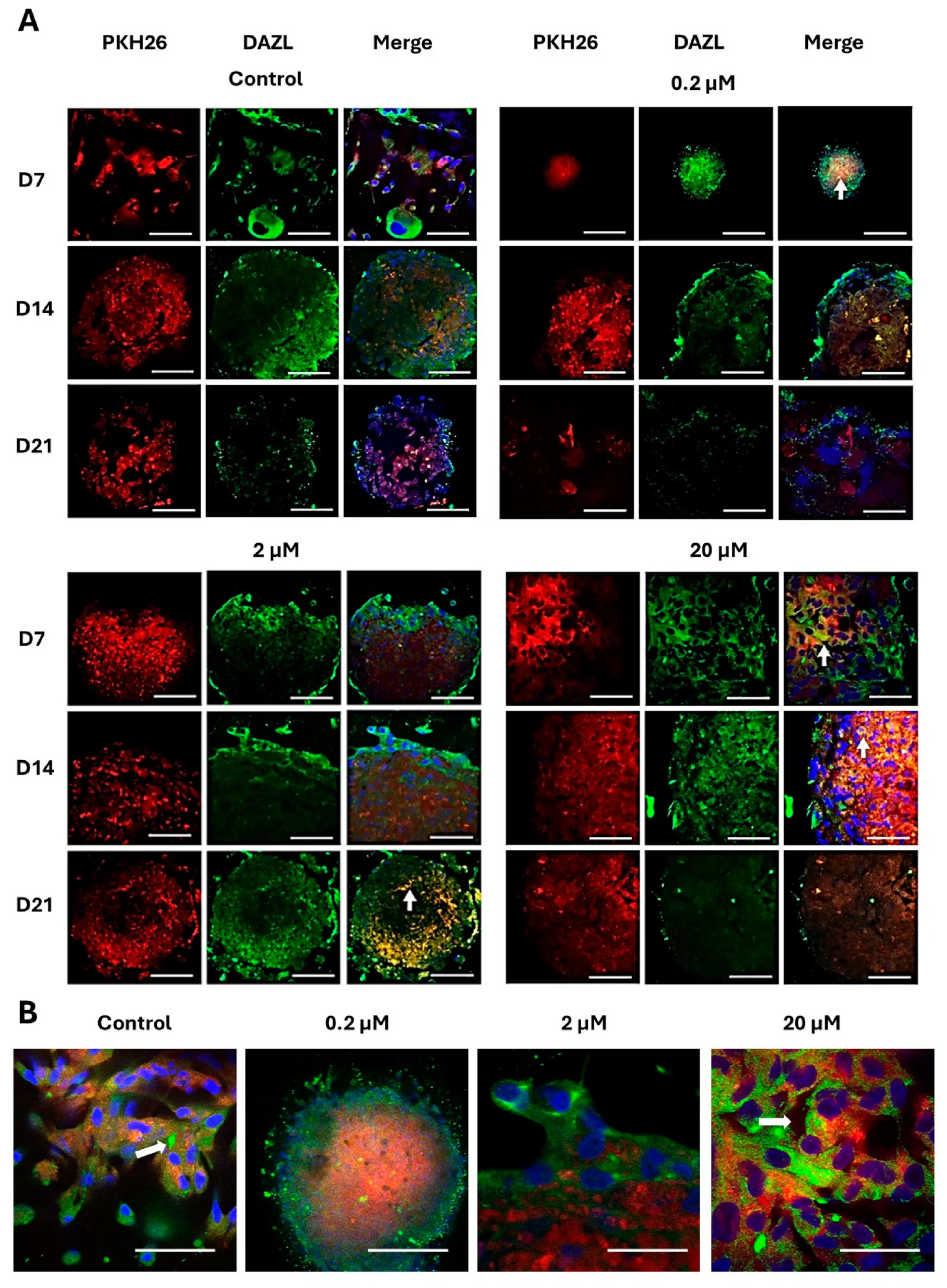
Localization of PKH26-positive AT-MSCs (Red) and DAZL (Green) expression in bovine TOs+AT-MSCs using confocal microscopy (7, 14 and 21 days after Matrigel seeding). (**A**) Control and TOs+AT-MSCs treated with 1 µM retinoic acid and 0.2, 2 and 20 µM of testosterone displayed a central and peripheral distribution pattern of DAZL-associated immunofluorescence during culture. (**B**) High-magnification evidence of spatial association between PKH26+ AT-MSCs and DAZL (white arrow). Cell nuclei were stained with DAPI (Blue). White arrow indicate overlapping red and green fluorescence. Scale bar: 100 μm. TOs (3) generated from each pool of cells (n = 3) were analyzed with confocal immunofluorescence for each day and treatment.

**Figure 4:**
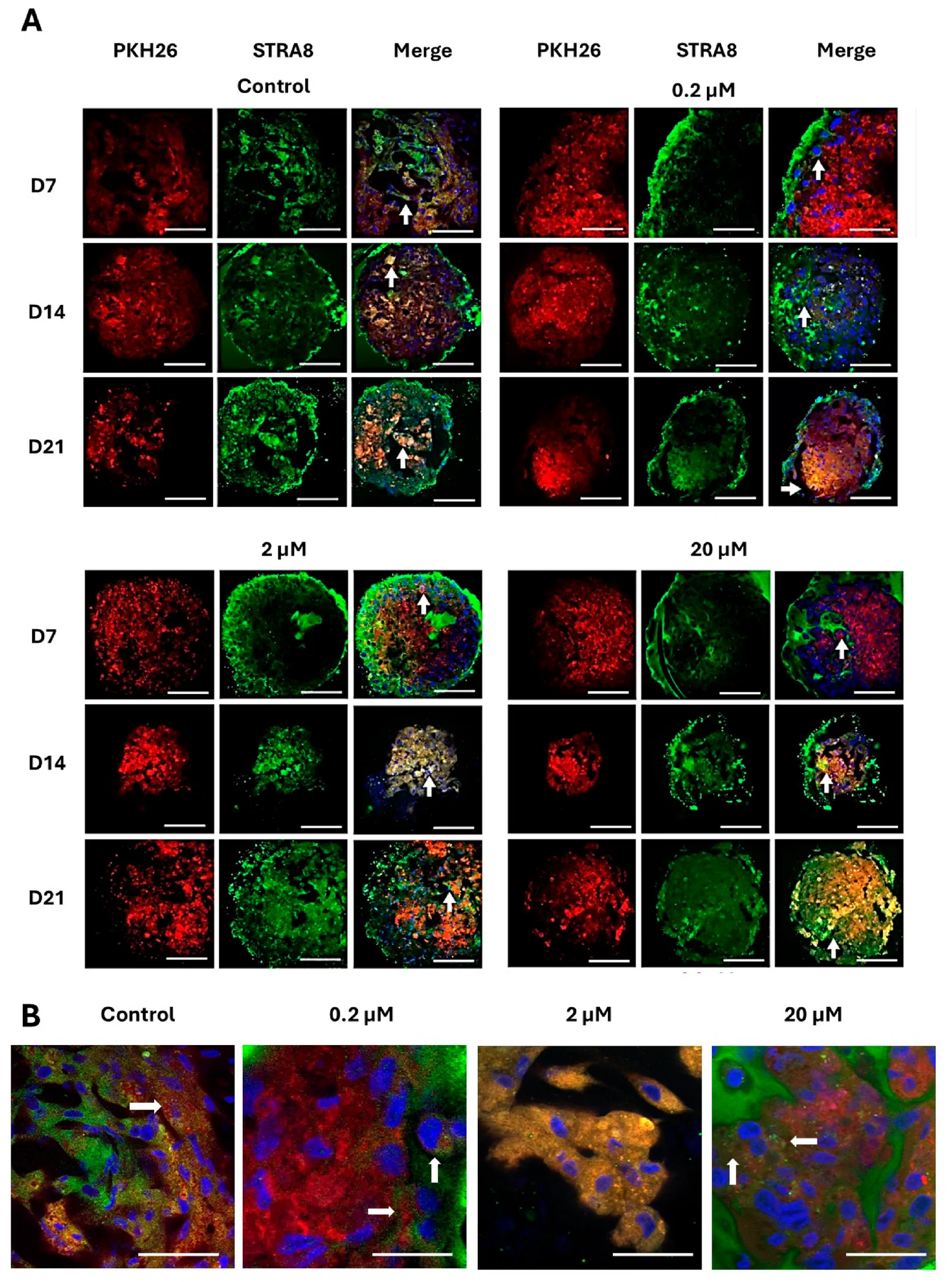
Localization of PKH26-positive AT-MSCs (Red) and STRA8 (Green) expression in bovine TOs+AT-MSCs using confocal microscopy (7, 14 and 21 days after Matrigel seeding). (**A**) STRA8 expression was observed with a predominantly peripheral pattern in control and TOs+AT-MSCs treated with retinoic acid and testosterone during the 21-day culture period. (**B**) High-magnification evidence of spatial association between PKH26+ AT-MSCs and STRA8 (white arrow). Cell nuclei were stained with DAPI (Blue). White arrow indicate overlapping red and green fluorescence. Scale bar: 100 μm. TOs (3) generated from each pool of cells (n = 3) were analyzed with confocal immunofluorescence for each day and treatment.

**Figure 5:**
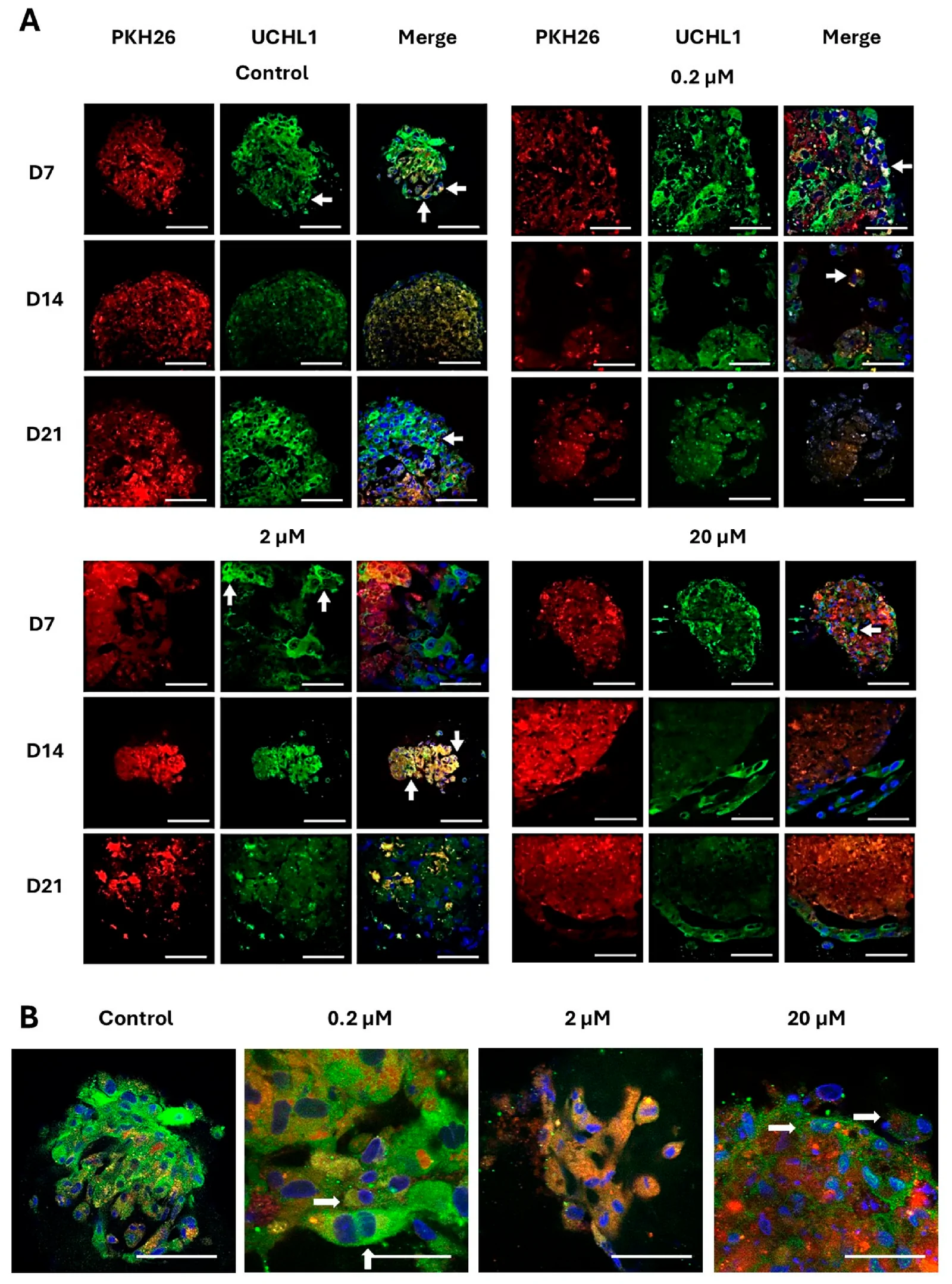
Localization of PKH26-positive AT-MSCs (Red) and UCHL1 (Green) expression in bovine TOs+AT-MSCs using confocal microscopy (7, 14 and 21 days after Matrigel seeding). (**A**) Immunosignal associated with UCHL1 was mainly localized in the central region of control and TOs+AT-MSCs treated with 1 µM of retinoic acid and all concentrations of testosterone during culture. (**B**) High-magnification evidence of spatial association between PKH26+ AT-MSCs and UCHL1 (white arrow). Cell nuclei were stained with DAPI (Blue). White arrow indicate overlapping red and green fluorescence. Scale bar: 100 μm. TOs (3) generated from each pool of cells (n = 3) were analyzed with confocal immunofluorescence for each day and treatment.

**Figure 6:**
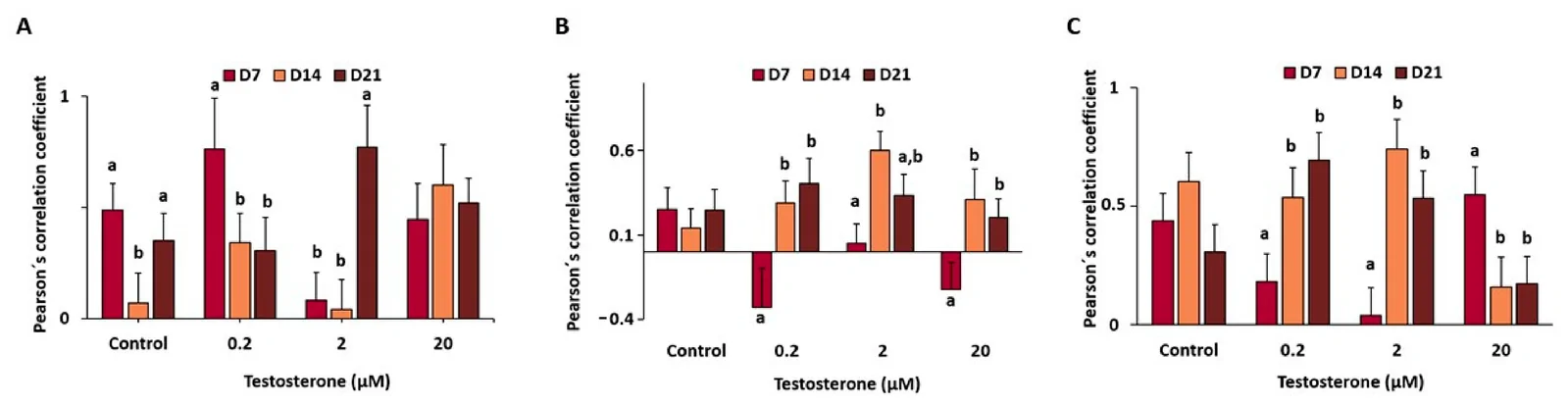
Pearson’s correlation coefficients for PKH26 and DAZL, STRA8 or UCHL1 in bovine TOs+AT-MSCs using confocal microscopy. (**A**) PCC values of PKH26 and DAZL were higher (*p* < 0.05) at days 7 and 21 in TOs+AT-MSCs treated with 1 µM retinoic acid and 0.2 or 2 µM of testosterone, respectively. (**B**) PCC values of PKH26 and STRA8 were lower (*p* < 0.05) at day 7 in TOs+AT-MSCs treated with 1 µM retinoic acid and 0.2, 2 or 20 µM of testosterone. (**C**) PCC values of PKH26 and UCHL1 were higher (*p* < 0.05) at days 14 and 21 in TOs+AT-MSCs treated with 1 µM retinoic acid and 0.2 or 2 µM of testosterone, respectively. Superscript and subscript (a, b) indicates significant (*p* < 0.05) difference in PCCs between days of culture (7, 14 and 21 days after Matrigel seeding) for each testosterone concentration. TOs (10–12) generated from each pool of cells (N = 3) were analyzed with PCC for each day and treatment.

A widespread distribution of PKH26-positive AT-MSCs was observed in the control and TOs+AT-MSCs treated with 1 µM of retinoic acid and 0.2, 2 and 20 μM of testosterone during the 21-day culture period (Figure 3, Figure 4 and Figure 5). Distributions of AT-MSCs in TOs+AT-MSCs were not affected by exogenous treatment with retinoic acid and different testosterone concentrations. The control and TOs+AT-MSCs treated with 1 µM retinoic acid and 0.2, 2 and 20 µM of testosterone displayed a central and peripheral distribution pattern of DAZL-associated immunofluorescence during culture (Figure 3). The PCC values of PKH26 and DAZL were lower (*p* < 0.05) in control TOs at day 14 (0.07 ± 0.13) compared to days 7 and 21 (Figure 6A). Moreover, PCC values were higher (*p* < 0.05) at days 7 (0.76 ± 0.23) and 21 (0.77 ± 0.18) in TOs+AT-MSCs treated with 1 µM retinoic acid and 0.2 or 2 µM of testosterone, respectively. STRA8 expression was observed with a predominantly peripheral pattern in the control and TOs+AT-MSCs treated with retinoic acid and testosterone during the 21-day culture period (Figure 4). The PCC values of PKH26 and STRA8 were lower (*p* < 0.05) at day 7 in TOs+AT-MSCs treated with 1 µM retinoic acid and 0.2 (−0.32 ± 0.2), 2 (0.05 ± 0.11) or 20 (−0.22 ± 0.16) µM of testosterone (Figure 6B). The immunosignal associated with UCHL1 was mainly localized in the central region of the control and TOs+AT-MSCs treated with 1 µM of retinoic acid and all concentrations of testosterone during culture (Figure 5). PCC values of PKH26 and UCHL1 were higher (*p* < 0.05) at days 14 and 21 in TOs+AT-MSCs treated with 1 µM retinoic acid and 0.2 (0.54 ± 0.23 and 0.7 ± 0.15) or 2 (0.74 ± 0.13 and 0.53 ± 0.08) µM of testosterone, respectively. However, the PCC value decreased (*p* < 0.05) in TOs+AT-MSCs treated with 1 µM retinoic acid and 20 µM of testosterone at days 14 (0.16 ± 0.18) and 21 (0.17 ± 0.11) (Figure 6C).

## 4. Discussion

Testicular organoids constitute an accessible mean to recreate cell-to-cell and cell-to-matrix interactions and to evaluate cell migration and differentiation [16,26,27]. Processes and methodologies employed for the production of TOs are highly diverse, ranging from variations in the number and types of testicular cells to differences in culture methods [20,22,26,27,28,29,30]. The protocol used in the present study comprises the isolation of testicular cells including Sertoli, Leydig and peritubular cells from bull testis and their subsequent co-culture with fetal bovine AT-MSCs during a 21-day culture period [24]. Previously, this protocol allowed us to develop TOs with the capacity for testosterone production and for tracking the migration of testicular and AT-MSCs [24]. In this model, inclusion of AT-MSCs resulted in consistently smaller diameters, compared to control TOs without AT-MSCs, throughout the culture period. In addition, TOs+AT-MSCs exhibited a more compact morphology, characterized by the absence of branching structures and a distinct peripheral cell layer [24]. Furthermore, in the present study, we used TOs to recreate the testicular niche under in vitro conditions with the aim of determining the spatial and temporal association of fetal bovine AT-MSCs with the expression of GC markers DAZL, STRA8, and UCHL1.

The isolated populations of AT-MSCs displayed high mRNA levels of MSC surface markers CD73, CD90, and CD105, along with low expression of hematopoietic markers CD34 and CD45, confirming characteristic MSC features. Comparable expression profiles of MSC and hematopoietic markers have previously been described in bovine fetal MSCs derived from peripheral blood (PB-MSCs) [10]. Analysis of the marker gene expression in testicular cells by Q-PCR indicate that the obtained cultures corresponded to specific testicular cell types including Sertoli cells (WT1), Leydig cells (STAR) and Peritubular myoid cells (αSMA). This data also corresponds to previous results reported by another group [26] and by our group [24] using the same protocol for testicular cell isolation, where each culture had populations of 21–37% of Leydig cells, 65–77% of Sertoli cells and 10–16% of peritubular myoid cells. Despite the protocol not achieving 100% separation of specific testicular cells types, it allowed us to obtain testicular cell-enriched fractions for the production of TOs. Furthermore, bovine Leydig, Sertoli, and peritubular myoid cells were combined at a concentration of 1 × 10^6^ cells/mL, maintained for 7 days, and subsequently transferred to Matrigel to promote 3D organization. This approach differs from commonly employed protocols in 3D testicular models, which typically rely on enzymatic digestion and removal of connective tissue to obtain mixed testicular cell populations [19,22,23]. Such conventional isolation procedures may inadvertently introduce additional cell types, including endothelial-like cells or other uncharacterized populations, which could interfere with downstream experiments [22,23]. Moreover, according to a previous report [31], our protocol reduced ~90% of endogenous GCs during TO formation, with the aim of decreasing endogenous GC marker expression during AT-MSC evaluation. However, residual GC may still be present in TOs+AT-MSCs.

We evaluated a protocol that included supplementation with exogenous retinoic acid and comparison of three concentrations of exogenous testosterone in TOs+AT-MSCs. Although we did not use separated controls to evaluate the individual effects of retinoic acid and testosterone, the combined effect of retinoic acid and testosterone has been reported to be crucial for testicular physiology, specifically, Sertoli cell function [32,33,34]. Retinoic acid, an active metabolite of vitamin A, and testosterone, an androgen hormone, are key components of the testicular microenvironment. In the present study, we found a significant reduction in the diameter of the testosterone-untreated control (day 21) and TOs+AT-MSCs treated with 0.2 µM (Day 14 and 21) compared to day 7. However, supplementation with retinoic acid and testosterone in concentrations of 2 or 20 µM resulted in a similar diameter in TOs+AT-MSCs at days 14 and 21 in comparison to day 7. The effect of exogenous retinoic acid and testosterone on the diameter of TO+AT-MSCs may be associated with increased proliferation of testicular cells. This potential proliferative outcome is speculative, and thus, other effects may account for the increased diameter of TOs+AT-MSCs, including extracellular matrix reorganization, structural stabilization, or differential cell survival; however further analyses are required to characterize these effects [25]. In the present study, testicular cells were isolated from post-pubertal testis, which corresponds to a less proliferative and more differentiative cell stage. Despite the reduced proliferative capacity of Sertoli cells in post-pubertal testis, Sertoli cells also express retinoic acid receptors (RARs) and respond to retinoic acid and testosterone combination by secreting growth factors involved in the control and maintenance of spermatogenesis in vivo [33]. Compared with Sertoli cells cultured without hormones, vitamins, or growth factors, Sertoli cells supplemented with testosterone and retinoic acid have been shown to upregulate post-meiotic genes and enhance stem cell differentiation capacity [34]. Alternatively, testosterone’s effect on the diameter of TOs may also be associated with increased proliferation of AT-MSCs. However, previous studies have reported that suppression of androgen receptor (AR) results in higher proliferation and self-renewal of AT-MSCs, which indicates that testosterone activity represses the proliferation of AT-MSCs [35]. Previously, we reported that TOs produced testosterone in concentrations of 0.17 to 0.31 ng/mL, which were lower compared to physiological total testosterone concentrations in plasma (5 to 8 ng/mL) [24]. In the present study, exogenous supplementation of retinoic acid and 2 or 20 µM of testosterone resulted in concentrations ~100- to 1000-fold higher compared to plasmatic levels. These concentrations of testosterone were extrapolated from previous in vitro GC differentiation studies in mouse MSCs [36] and ESCs [37] and bovine SSCs [38]. Over physiological concentrations are normally used in in vitro studies to compensate for the absence of pharmacokinetics, tissue accumulation, distribution and synergy systems, and for the reduced plasma protein binding, sensitivity and exposure time [39]. However, these exogenous supplementation levels were more similar to testicular testosterone concentrations, which are 25–125-fold higher in bovine testis compared to the periphery [40,41]. These results suggest that in vitro conditions including retinoic acid supplementation and concentrations of testosterone over physiological levels may induce the proliferation of testicular cells that maintain the diameter of TO+AT-MSCs after 7 days of culture.

Using a confocal microscopy analysis, we observed that DAZL expression was present in the TOs+AT-MSCs control and TOs+AT-MSCs treated with retinoic acid and testosterone during the 21-day culture period. The PKH26-positive AT-MSCs location coincided with DAZL expression in TOs+AT-MSCs at several stages; however, higher PCC values of PKH26- and DAZL-positive cells were observed at day 21 in TOs+AT-MSCs treated with 1 µM of retinoic acid and 2 µM of testosterone compared to day 7 and 14. DAZL is essential for mammalian spermatogenesis and is involved in the proliferation, development, maturation, and functional maintenance of male GCs [42,43,44]. DAZL is expressed in the peripheral areas of seminiferous tubules mainly associated with pre-spermatogonial cells [42] but is also present in Leydig cells [43] and bovine MSCs [10]. In comparison, expression of STRA8 was detected in the control and TOs+AT-MSCs treated with 1 µM of retinoic acid and all concentrations of testosterone during the 21-day culture period. Moreover, higher PCC values of PKH26- and STRA8-positive AT-MSCs were detected in TOs+AT-MSCs treated with 1 µM of retinoic acid and 0.2 and 20 µM testosterone at later days of culture (days 14 and 21). STRA8 is considered a specific GC marker, whose function is regulated by retinoic acid and controls the entrance of GCs into meiosis [45]. Retinoic acid favors spermatogonial differentiation through a direct action and an indirect effect mediated by BMP4 secreted by Sertoli cells [45]. STRA8 expression is observed in the basal compartment of the germinal epithelium in seminiferous tubules but has also been detected in bovine AT-MSCs [42]. Furthermore, when analyzing the immunosignal associated with UCHL1, it was observed in the control and TOs+AT-MSCs treated with retinoic acid and all concentrations of testosterone during all culture stages analyzed. Higher PCC values of PKH26- and UCHL1-positive AT-MSCs were detected in TOs+AT-MSCs treated with 1 µM of retinoic acid and 0.2 or 2 µM of testosterone at later days of culture (days 14 and 21) compared to day 7. However, treatment with the highest concentration of testosterone (1 µM of retinoic acid and 20 µM of testosterone) reduced PCC at days 14 and 21. We have not characterized the specific location of UCHL1 in bovine testis; however, previous studies have reported that UCHL1 is consistently and specifically expressed in bull SSCs [10,46,47]. UCHL1 is expressed in male premeiotic GCs and shows no affinity for somatic testicular cells, making it an optimal marker for spermatogonia in domestic bull testes [46]. However, we have previously reported the expression of UCHL1 in bovine PB-MSCs [47]. Thus, considering the different testicular cells types composing TOs+AT-MSCs, immunopositivity to DAZL and UCHL1 may be associated with respective expression in Leydig and Sertoli cells. Moreover, considering that the cell isolation protocol used in the present study eliminated most of the GCs (~90%) derived from testicular extracts [31], the expression associated with STRA8, and in some proportion DAZL and UCHL1, may also be a consequence of residual GCs present in TOs+AT-MSCs. Nevertheless, as previously reported by our group for 2D culture models where these GC markers were detected in AT-MSCs [10,42,46], immunodetection of DAZL, STRA8 and UCHL1 in TOs+AT-MSCs may also be associated with expression in AT-MSCs. Overall, our qualitative and quantitative analyses revealed a spatial and temporal association between AT-MSC localization and GC marker immunosignals in TOs+AT-MSCs treated with 1 µM retinoic acid and 2 µM of testosterone. However, higher PCCs positive for STRA8, and UCHL1 were also detected at different days in TOs+AT-MSCs treated with 1 µM retinoic acid and 0.2 µM of testosterone.

Despite the wide use of PKH26 as a cell tracking dye in in vivo cell transplantation trials and organoid experiments [48,49], unspecific staining due to dye transfer to unlabeled cells and dilution in proliferating cells has been reported [50]. In the present study, transgenic cell lines with more specific reporters were not available. Although PKH26 possess limitations, this dye has been previously used by our group as a simple and less expensive tracking system for AT-MSCs in TOs. Further analyses are required to determine the specificity of PKH26 in organoids and specifically in TO research.

## 5. Conclusions

Exposure to retinoic acid and concentrations of testosterone over physiological levels under in vitro conditions resulted in similar diameters of TO+AT-MSCs after 7 days of culture. Spatial associations between AT-MSC localization and GC marker immunosignals were mainly detected in TOs+AT-MSCs treated with 1 µM retinoic acid and 2 µM of testosterone.

## Figures and Tables

**Table 1:** Primer sequences used in Q-PCR analyses.

Gene	Nucleotide Sequence (5′-3′)		Access Number
β-ACTIN	Forward Reverse	CGCACCACTGGCATTGTCAT TCCAAGGCGACGTAGCAGAG	NM_173979.3
GAPDH	Forward Reverse	CCTTCATTGACCTTCACTACATGG TCTA TGGAAGATGGTGATGGCCTTTCCATTG	NM_001034034.2
CD73	Forward Reverse	TGGTCCAGGCCTATGCTTTTG GGGATGCTGCTGTTGAGAAGAA	BC114093
CD90	Forward Reverse	CAGAATACAGCTCCCGAACCAA CACGTGTAGATCCCCTCATCCTT	BC104530
CD105	Forward Reverse	CGGACAGTGACCGTGAAGTTG TGTTGTGGTTGGCCTCGATTA	NM 001076397
CD34	Forward Reverse	TGGGCATCGAGGACATCTCT GATCAAGATGGCCAGCAGGAT	AB021662
CD45	Forward Reverse	CCTGGACACCACCTCAAAGCT TCCGTCCTGGGTTTTATCCTG	NM 001206523
αSMA ***	Forward Reverse	CAGCCGAGAACTTTCAGGGAC GGTGATGATGCCGTGCTCTA	NM_001034502.1
STAR **	Forward Reverse	GACACGGTCATCACTCACGA TACGCTCACAAAGTCTCGGG	NM_174189.3
WT1 *	Forward Reverse	ACAGATGCACAGCCGGAAGC GGTGGTCGGAACGGGAGAA	XM_015470745.2

* Wilm’s Tumor 1; ** Steroidogenic acute regulatory; *** Actin alpha 2, smooth muscle (α-SMA).

**Table 2:** Primary and secondary antibodies used for AT-MSCs and testicular cells immunodetection.

Antibody	Target Cell	P/N	Cat.	Dilution
Anti-DAZL	GC	Abcam	ab34139	1:100
Anti-STRA8	GC	Abcam	ab49602	1:100
Anti-UCHL1	SSC	Thermo-Fisher	480012	1:100
Anti-mouse	Secondary	Thermo-Fisher	A11001	1:100
Anti-rabbit	Secondary	Abcam	ab97050	1:100

## Data Availability

The original contributions presented in this study are included in the article/Supplementary Material. Further inquiries can be directed to the corresponding author.

## Supplementary Materials

The following supporting information can be downloaded at: https://www.mdpi.com/article/10.3390/ani16182933/s1, Figure S1. Negative controls for confocal immunofluorescence analysis of DAZL, STRA8 and UCHL1 in TOs+AT-MSCs; File S1. Diameter and PCC Data.
