# Supplementary material for: Associations of Bovine Fetal Adipose Tissue-Derived Mesenchymal Stem/Stromal Cells and Germ Cell Marker Expression in Testicular Organoids
Source: Animals (Basel). 2026 Sep 18;16(18):2933. doi: 10.3390/ani16182933 (PMC13603360; doi:10.3390/ani16182933)
Supplement: Supplementary file 1 [file animals-16-02933-s001.zip › Figure S1 (Negative Controls).pdf]

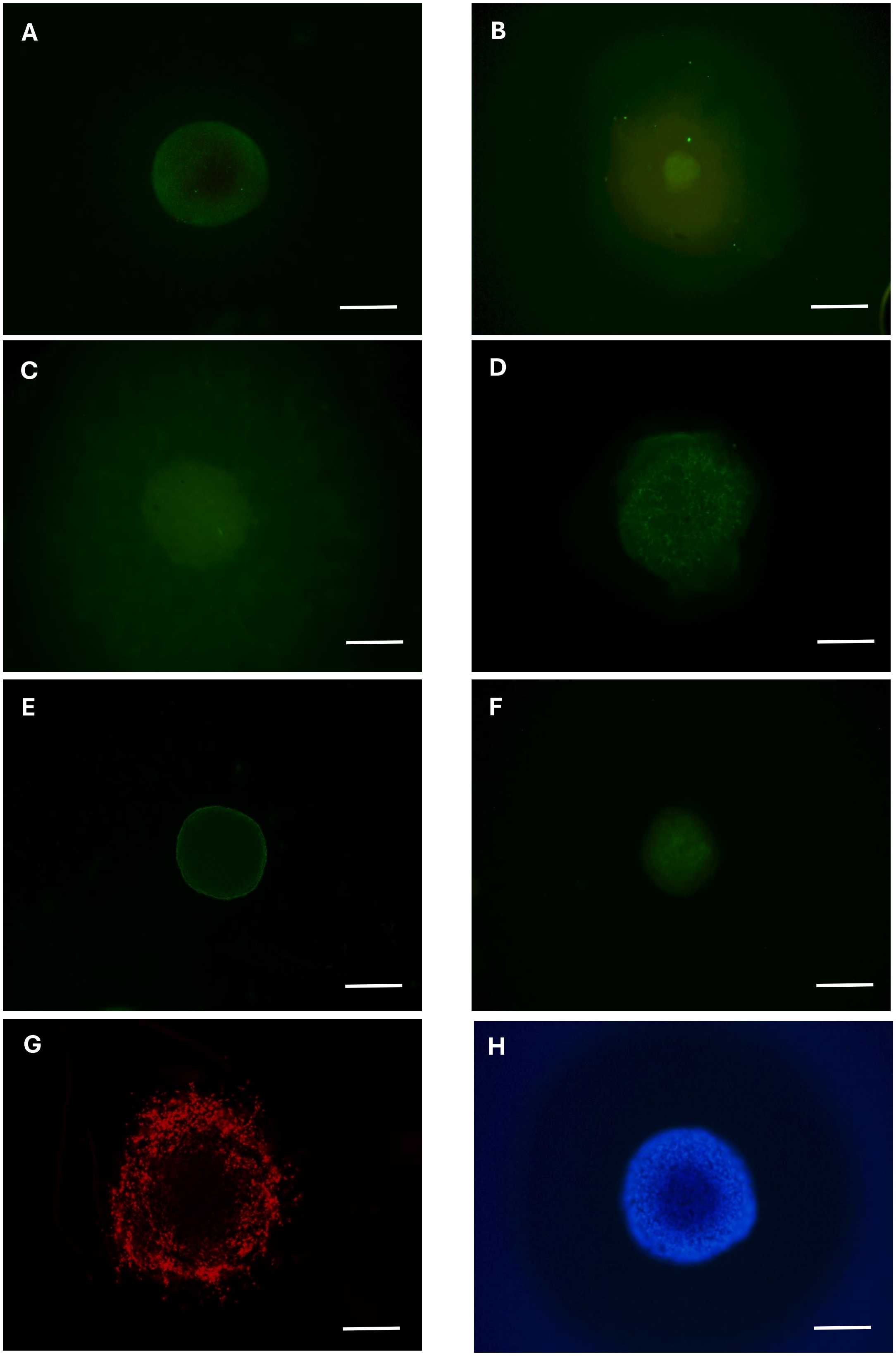
A
B
C
D
E
F
G
H

Figure S1. Negative controls for confocal immunofluorescence analysis of DAZL, STRA8 and UCHL1 in TOs+AT-MSCs. Negative controls consisted in replacement of the primary antibody with non-immune serum for DAZL (A), STRA8 (B) and UCHL1 (C) immunodetection. Negative controls also consisted in omission of both primary and secondary antibodies for DAZL (D), STRA8 (E) and UCHL1 (F) immunodetection. Representative images of PKH26 (G) and DAPI (H) fluorescence. Antibodies: Anti-DAZL (Abcam, ab34139, 1:100); Anti-STRA8 (Abcam, ab49602, 1:100); Anti-UCHL1 (Thermo-Fisher, 480012, 1:100). Scale bar: 100 μm.
